# High-throughput diagnostic markers for foliar fungal disease resistance and high oleic acid content in groundnut

**DOI:** 10.1186/s12870-024-04987-9

**Published:** 2024-04-10

**Authors:** Manish K. Pandey, Sunil S. Gangurde, Yaduru Shasidhar, Vinay Sharma, Sandip M. Kale, Aamir W. Khan, Priya Shah, Pushpesh Joshi, Ramesh S. Bhat, Pasupuleti Janila, Sandip K. Bera, Rajeev K. Varshney

**Affiliations:** 1 International Crops Research Institute for the Semi-Arid Tropics (ICRISAT), Hyderabad, India; 2 University of Agricultural Sciences, Dharwad, India; 3 ICAR-Directorate of Groundnut Research, Junagadh, India; 4 Centre for Crop and Food Innovation, WA State Agricultural Biotechnology Centre, Murdoch University, Murdoch, Australia

**Keywords:** Late leaf spot, Leaf rust, Gene-based markers, Candidate gene discovery, Diagnostic markers

## Abstract

**Background:**

Foliar diseases namely late leaf spot (LLS) and leaf rust (LR) reduce yield and deteriorate fodder quality in groundnut. Also the high oleic acid content has emerged as one of the most important traits for industries and consumers due to its increased shelf life and health benefits.

**Results:**

Genetic mapping combined with pooled sequencing approaches identified candidate resistance genes (*LLSR1* and *LLSR2 *for LLS and *LR1* for LR) for both foliar fungal diseases. The LLS-A02 locus housed *LLSR1* gene for LLS resistance, while, LLS-A03 housed *LLSR2* and *LR1* genes for LLS and LR resistance, respectively. A total of 49 KASPs markers were developed from the genomic regions of important disease resistance genes, such as NBS-LRR, purple acid phosphatase, pentatricopeptide repeat-containing protein, and serine/threonine-protein phosphatase. Among the 49 KASP markers, 41 KASPs were validated successfully on a validation panel of contrasting germplasm and breeding lines. Of the 41 validated KASPs, 39 KASPs were designed for rust and LLS resistance, while two KASPs were developed using fatty acid desaturase (FAD) genes to control high oleic acid levels. These validated KASP markers have been extensively used by various groundnut breeding programs across the world which led to development of thousands of advanced breeding lines and few of them also released for commercial cultivation.

**Conclusion:**

In this study, high-throughput and cost-effective KASP assays were developed, validated and successfully deployed to improve the resistance against foliar fungal diseases and oleic acid in groundnut. So far deployment of allele-specific and KASP diagnostic markers facilitated development and release of two rust- and LLS-resistant varieties and five high-oleic acid groundnut varieties in India. These validated markers provide opportunities for routine deployment in groundnut breeding programs.

**Supplementary Information:**

The online version contains supplementary material available at 10.1186/s12870-024-04987-9.

## Introduction

Groundnut (*Arachis hypogaea* L.), or peanut, is cultivated in more than 100 countries worldwide covering∼33.2 million hectares with an annual production of 72.3 million tons [1]. A total of 100 g of groundnut oil contained 17.7 g of saturated fat, 48.3 g of monounsaturated fat (primarily oleic acid), and 33.4 g of polyunsaturated fat (comprising linoleic and linolenic acid). High oleic acid increases the shelf life of groundnut oil and has important health benefits [2]. The popularity of groundnut oil in Chinese, South Asian and Southeast Asian countries is because of its high smoke point, making it ideal for preparation of fried food delicacies.

Foliar fungal diseases, namely, rust caused by *Puccinia arachidis* and late leaf spot (LLS) caused by *Nothopassalora personata*, are highly destructive when they occur together, leading to substantial yield losses of 50–70%, as well as deterioration of fodder quality [3]. The rate of photosynthesis decreases as the healthy leaf area decreases due to the large number of leaf spots and defoliation. In addition to yield loss and reduced fodder quality, farmers bear the extra cost of fungicide application which impacts both their income and environmental health. As a result, developing groundnut varieties with rust and LLS resistance is a key objective for groundnut breeding programs worldwide to sustain productivity .

The marker-assisted backcrossing (MABC) approach has been successfully used to improve resistance to rust and late leaf spot as well as to increase the oleic acid content in groundnut varieties [4]. There has been successful marker deployment in groundnut breeding using low-throughput genotyping assays such as simple sequence repeats (SSRs) and allele-specific markers for improving foliar disease resistance [2, 5–8] and high oleic acid content [2, 8–12]. The deployment of available allele-specific markers and KASP (Kompetitive Allele Specific PCR) assays developed under this study have already resulted in the commercial cultivation of five high-oleic acid varieties in five Indian states (Gujarat, Rajasthan, Karnataka, Andhra Pradesh, Telangana and Tamil Nadu). The two Virginia Bunch varieties, Girnar 4 (ICGV 15083) and Girnar 5 (ICGV 15090) were developed from the cross ICGV 06420 × (ICGV 06420 × Sun Oleic 95-R) while the three Spanish Bunch varieties, GG 39 (ICGV 16697), ICRC-1 (ICGV 16690) and GG 40 (ICGV 16668), were developed from the cross ICGV 06110 × (ICGV 061l0 × Sun Oleic 95-R); and the high oleic acid donor SunOleic 95R [9]. In addition, there are several promising marker-assisted bred lines (ICGV 181023, ICGV 181025 and ICGV 171025) in the genetic background of three elite varieties, ICGV 06142, ICGV 06420 and ICGV 061l0 [9] which are under third-year testing in the All India Coordinated Research Project on Groundnut (AICRP-G). Furthermore, two foliar disease-resistant varieties, namely, Improved JL 24 (DBG 3) and Super TMV 2 (DBG 4) have also been developed using the resistant donor GPBD 4 [6, 7] and released in the state of Karnataka, India for commercial cultivation. The low-throughput markers such as allele-specific and SSR markers are good for genotyping small number of breeding lines, however, genotyping and selection in large scale breeding samples require high-throughput genotyping technologies to perform selection in time. Further, the availability of high-throughput markers such as KASP assays provide an opportunity to perform selection for multiple traits which benefit breeding teams in saving resources and to stack multiple traits.

With recent advancements in sequencing technologies, high-throughput genotyping has become more feasible, facilitating the identification of associated genomic regions and candidate genes and the development of diagnostic markers for use in early-generation selection in several crops, including groundnut [4, 13, 14]. In addition to the already available reference genomes of diploids [15–17], the further availability of the high-density genotyping assay “Axiom_*Arachis*” with 58 K SNPs [18, 19] and high-quality reference genomes [20–22] for both subspecies of cultivated tetraploid groundnut further enhances trait dissection and marker development for target traits in groundnut.

Previous studies using a recombinant inbred line (RIL) population developed from the cross TAG 24 and GPBD 4 identified major quantitative trait loci (QTLs) for rust resistance on chromosome A03 and two major QTLs for LLS resistance on chromosomes A03 and A02 [23–25]. The QTL for rust explained up to 82.6% of the phenotypic variance (PVE), while both QTLs for LLS resistance explained 40–60% PVE; the A03 genomic region is common for both diseases, rust and LLS [23]. SSR markers linked to these traits have been developed and used for the introgression of rust and LLS resistance [2, 5]. However, genotyping with the currently available linked SSR markers for these traits is time consuming and laborious, hindering their large-scale adoption in breeding programs. Marker-assisted early generation selection (MEGS) has emerged as an effective approach for selecting desired alleles in early generations, such as F_2_ in groundnut [4, 26]. However, MEGS requires diagnostic markers with high selection efficiency so that decisions can be made at very early stages of the breeding program. Therefore, this study reports the development of low-density and high-throughput single nucleotide polymorphism (SNP)-based KASP genotyping assays for two foliar fungal diseases and high oleic acid content in groundnut. These markers are now routinely used for tracking desirable alleles for these traits in breeding populations and germplasms.

## Materials and methods

### Genetic mapping population and validation panel

The RIL mapping population (TAG 24 × GPBD 4) comprising 266 individuals was developed at the University of Agricultural Sciences-Dharwad (UAS-Dharwad), India. The resistant parent, GPBD 4, is an elite groundnut variety used as a national check, derived from the cross KRG 1 × ICGV 86855 (CS 16). Notably, ICGV 86855 (CS 16), an interspecific derivative of *A. cardenasii*, is a source of rust and LLS resistance [27]. The RIL population (TAG 24 × GPBD 4) was phenotyped extensively for both foliar fungal diseases, i.e., leaf rust and LLS, at the University of Agricultural Sciences, Dharwad, India, and the details are available in our previous studies [23–25]. Multiple seasons of phenotyping data have been generated on the RIL population TAG 24 × GPBD 4 for leaf rust and LLS resistance for seven years/seasons (2004 to 2010) and reported in previous studies with SSR [23] and RAD-Seq to identify the QTLs associated with rust and LLS resistance. The disease rating of the RIL population was performed at 80 days after sowing (DAS) and 90 DAS for rust resistance and at 70 DAS and 90 DAS for LLS resistance [23]. Further details on the phenotyping procedure for rust and LLS have been provided by Sujay et al. [23]. The extreme resistant and susceptible individuals from the RIL population (TAG 24 × GPBD 4) based on phenotyping data were used for developing two DNA bulks for sequencing and QTL-seq analysis to identify the genomic regions and candidate genes for leaf rust and late leaf spot resistance in groundnut [22, 24].

We used separate validation panels individually for each LLS, rust and high oleic acid content. The validation panel for LLS included 20 lines (10 resistant and 10 susceptible), 43 for rust (21 resistant and 22 susceptible) and 51 for oleic acid (25 high oleic acid and 26 with low oleic acid content). The sources of high oleic acid, SunOleic 95R, as well as the derived high oleic acid varieties Girnar 4 (ICGV 15083), Girnar 5 (ICGV 15090), GG 40 (ICGV 16688) and ICRC-1 (ICGV 16690) from SunOleic 95R, were also included in the validation panel. The abovementioned genotype sets were used for the validation of KASP markers and correlated with preliminary phenotypic data. Furthermore, based on accuracy, highly efficient KASP markers were identified for breeding purposes.

### Identification of QTLs for rust and LLS resistance

#### Genotyping by sequencing of the RIL population

DNA from 217 RILs and the two parents was extracted using a Nucleospin Plant II kit (Macharey-Nigel, Duren, Germany). The DNA quality and quantity were checked on 0.8% agarose and then with a Qubit 2.0 fluorometer (Thermo Fisher Scientific Inc., USA). Low-coverage sequencing (GBS: genotyping-by-sequencing) was performed for the entire RIL population for simultaneous SNP discovery. A total of 10 ng of DNA from each RIL was digested using the restriction enzyme *ApeKI* endonuclease, which recognizes the G/CWCG site. The ligation enzyme *T4 ligase* was used to ligate the digested products with uniquely barcoded adapters. Such digestion and ligation were performed for each RIL, and then equal proportions from each sample were mixed to construct the libraries. These libraries were then amplified and purified to remove excess adapters. These DNA libraries were then sequenced on the HiSeq 2500 platform (Illumina Inc., San Diego, CA, USA) to generate genome-wide sequence reads at Center of Excellence in Genomics and Systems Biology (CEGSB), ICRISAT, Hyderabad, India. The sequence raw reads in FASTQ files generated for the RIL population and parental genotypes were used for SNP discovery using TASSEL v4.0 [28]. The groundnut progenitor (*A. ipaensis* and *A. duranensis*) genomes were used as reference assemblies for SNP calling [15].

#### Genetic mapping using a GBS-based genetic map

The GBS data generated from 217 RILs and two parental genotypes (TAG 24 and GPBD 4) were used for SNP discovery. Diploid progenitor genome assemblies (A- and B- subgenome) were used as reference assemblies for SNP calling. The chi-square (χ2) values calculated for each SNP marker were used to determine the goodness of fit to the expected 1:1 segregation ratio; highly distorted markers were filtered out and not included in the genetic map. The dense genetic map was constructed using JoinMap version 4 [29] and was redrawn using MapChart [30]. The Kosambi map function [31] with a recombination frequency of 0.45 was used to determine the map order for these new SNP markers by keeping the order fixed for earlier mapped SSR marker loci [23]. QTL analysis was performed using the composite interval mapping model implemented in Win-QTL cartographer 2.5 software [32].

#### Identification of genomic regions for rust and LLS resistance using QTL-Seq

For QTL-Seq analysis, DNA from 25 RILs with high susceptibility scores for rust (Rust_Sbulk) and LLS (LLS_Sbulk) but high resistance scores for rust (Rust_Rbulk) and LLS (LLS_Rbulk) were pooled separately to construct sequencing bulks. QTL-Seq analysis was performed using diploid [24] and tetraploid [22] genomes (Supplementary Table 1). These libraries generated 250-base paired-end reads on the Illumina HiSeq 2500 platform and the analysis was performed using the QTL-Seq pipeline (http://genome-e.ibrc.or.jp/home/bioinformatics-team/QTL-seq).

### Candidate gene discovery and marker development

The discovery of candidate genes and allele-specific markers was performed from the QTL/genomic regions identified in three different analyses, namely, (a) GBS-based genetic mapping and QTL analysis; (b) QTL-Seq analysis using a synthetic tetraploid reference genome built from individual diploid genomes for the A- and B-sub-genomes (genomes reported by Bertioli et al. [15]; and (c) QTL-Seq analysis using a tetraploid reference genome for the *fastigiata* subspecies of the cultivated groundnut genome [22]. Initially, low-throughput and easy-to-use allele-specific markers were developed and validated. The validated markers were converted into low-cost and high-throughput genotyping assays, also called KASP assays, for large-scale and easy deployment in breeding programs. To make this assay more valuable, KASP markers for high oleic acid content from fatty acid desaturase genes (*FAD2B* and *FAD2A*) have also been included in the genotyping assay for pyramiding high oleic acid content and foliar disease resistance. KASP assays were validated on diverse sets of resistant and susceptible genotypes and deployed successfully in developing marker-assisted backcrossed introgression lines [2, 33].

For high oleic acid, the high-oleic acid varieties Girnar 4 and Girnar 5 were sequenced at 10X coverage using an Illumina HiSeq2500 platform. Subsequently, the obtained sequences were aligned to the reference genome to identify non-synonymous SNPs in the *FAD2A* and *FAD2B* genes. Next, we developed allele-specific genotyping assays to accurately select both mutant alleles.

### Development and validation of a high-throughput genotyping assay for foliar fungal disease (rust and late leaf spot) resistance and high oleic acid content

SNPs from QTL regions, specifically from coding regions and affecting the function of important disease resistance-related candidate genes, were used for the development of KASP assays. These SNPs were selected from the regions 1 kb upstream or downstream or ideally within candidate genes present in the QTL regions on chromosomes A02 (LLS resistance) and A03 (rust and LLS resistance) using mapping information from GBS analysis and B03 (rust and LLS resistance) using information from QTL-seq analysis. Additionally, SNPs from genic regions of the fatty acid desaturase (*FAD*) gene from both sub-genomes, *A. ipaensis* (*fad2a*) on chromosome Ah09 and *A. duranensis* (*fad2b*) on Ah19, were targeted to design KASP markers for high oleic acid content. The SNPs were converted into KASP markers by using 50 bp upstream and 50 bp downstream sequences to develop user-friendly and cost-effective markers. KASP assays for each SNP marker were designed with two allele-specific forward primers and a common reverse primer. All the KASP primers used in this study are listed in Supplementary Table 2. Following development, the KASP markers were further validated on a diverse validation panel comprising both resistant and susceptible genotypes for rust and LLS as well as low- and high-oleic acid breeding lines.

## Results

### Genomic regions for leaf rust and LLS resistance

A total of 50 Gb of clean read data were generated for 217 RILs and two parental genotypes (TAG 24 and GPBD 4) of the mapping population. Additionally, resistant and susceptible bulks were constructed by mixing pooled DNA from 25 RILs exhibiting extreme phenotypes, i.e. resistant and susceptible for leaf rust (mean disease score of 3.7 for the resistant bulk and 7.7 for the susceptible bulk) and LLS (mean disease score of 4.4 for the resistant bulk and 8.1 for the susceptible bulk, as explained in [24]). The susceptible parent, TAG 24, had mean disease scores of 7.5 and 8.4 for rust and LLS, respectively, while the resistant parent, GPBD 4, had scores of 3.0 and 3.7, respectively.

**Fig. 1:**
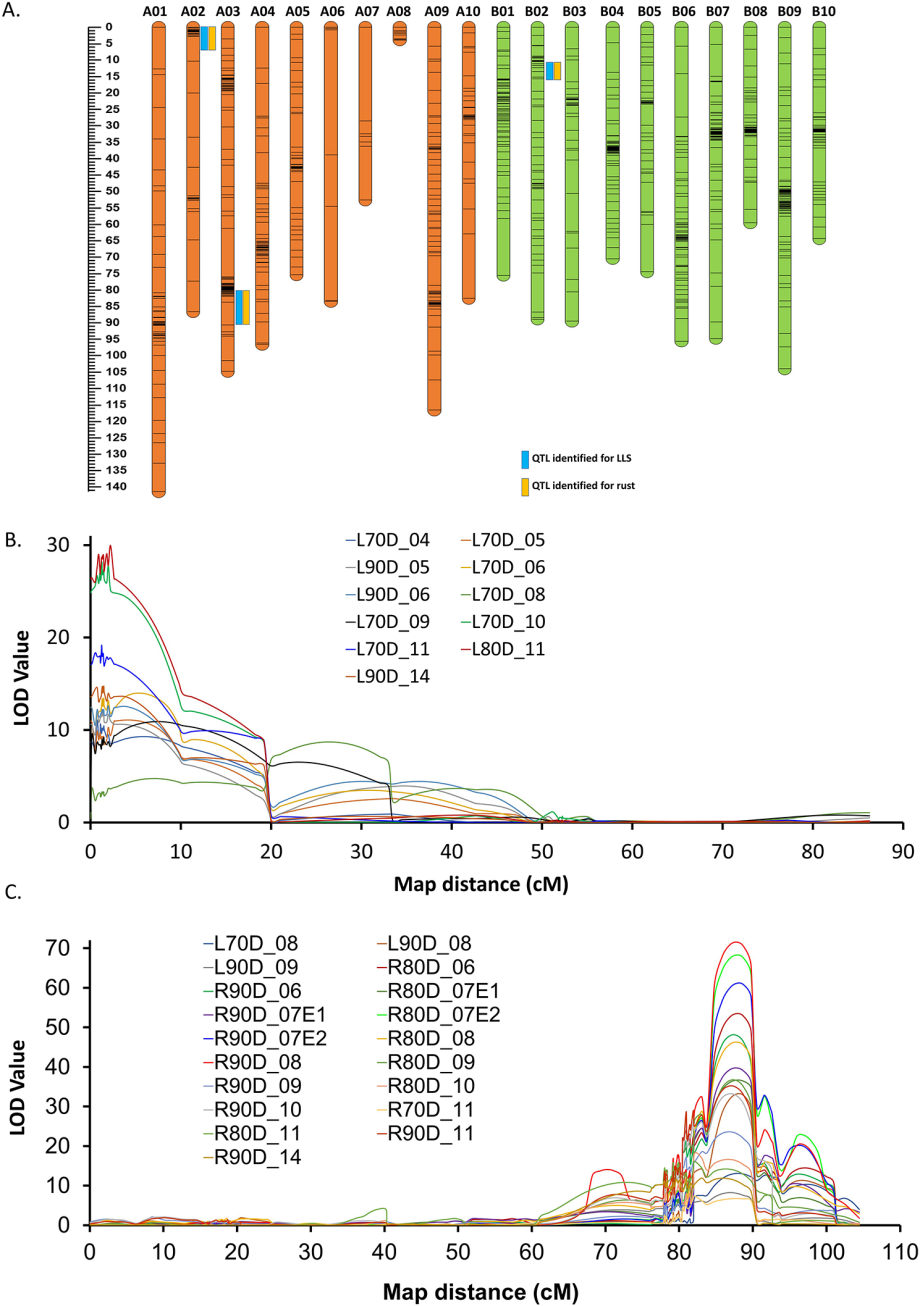
Genetic map and major QTLs identified for leaf rust and late leaf spot resistance. (**A**) Genetic map constructed using genotyping-by-sequencing data generated on the RIL population TAG 24 × GPBD 4. (**B**) Consistent major effect QTL identified on chromosome A02. (**C**) Consistent major effect QTL identified on chromosome A03

A total of 53,103 single nucleotide polymorphisms (SNPs) were identified across the RIL population using genotyping-by-sequencing. After filtration, 1,529 SNPs were found to be polymorphic between the parents. The chi-square (χ2) values calculated for each SNP marker were used to determine the goodness of fit to the expected 1:1 segregation ratio; highly distorted and unlinked markers were filtered out and not considered for linkage map construction. A dense genetic map with 1,119 SNP loci, including 831 SNPs and 288 simple sequence repeats (SSRs), was constructed with a map density of 1.48 cM/loci and a map length of 1,660.5 cM (Fig. 1A; Table 1).

**Table 1:** Major QTLs identified for leaf rust and late leaf spot resistance in groundnuts using the RIL population TAG 24 × GPBD 4

SN	QTL name	Trait	Year	Chromosome	Marker interval	Position (cM)	LOD	PVE (%)	Add effect
1.	*qRust-A02.1*	Rust_70	D14	A02	S2_464902 - S2_768713	1.2	11.3	30.6	-0.5
2.	*qLLS-A02.1*	LLS_90	D14	A02	S2_880921 - S2_99621	0.7	14.6	23.3	-0.8
		LLS_70	D11	A02	S2_880921 - S2_99621	0.5	18.3	30.1	-0.2
3.	*qLLS-A02.2*	LLS_90	D05	A02	S2_976663 - S2_454537	2.0	11.7	16.3	-0.6
		LLS_70	D10	A02	S2_976663 - S2_454537	2.0	27.8	40.2	-0.5
		LLS_80	D11	A02	S2_976663 - S2_454537	2.2	30.0	44.2	-0.5
4.	*qLLS-A02.3*	LLS_70	D04	A02	S2_1228446 - S2_1281741	5.7	9.3	17.3	-0.5
		LLS_70	D05	A02	S2_1228446 - S2_1281741	3.7	11.1	17.5	-0.6
		LLS_90	D06	A02	S2_1228446 - S2_1281741	3.7	12.6	19.0	-0.6
		LLS_70	D06	A02	S2_1228446 - S2_1281741	5.7	14.0	23.9	-0.7
5.	*qLLS-A02.4*	LLS_70	D09	A02	S2_1240859 - S2_1209628	0.1	9.4	15.2	-0.4
		LLS_70	D05	A02	S2_1240859 - S2_1209628	0.0	11.0	16.1	-0.6
		LLS_70	D06	A02	S2_1240859 - S2_1209628	0.0	12.4	18.2	-0.6
		LLS_70	D04	A02	S2_1240859 - S2_1209628	0.1	8.7	14.7	-0.5
		LLS_70	D08	A02	S2_1240859 - S2_1209628	0.1	3.7	5.1	-0.2
		LLS_90	D04	A02	S2_1240859 - S2_1209628	0.0	4.4	7.1	-0.4
6.	*qLLS-A02.5*	LLS_70	D06	A02	S2_2024918 - S2_4433397	31.0	3.5	5.6	-0.3
		LLS_70	D08	A02	S2_2024918 - S2_4433397	40.5	3.7	6.0	-0.2
		LLS_90	D05	A02	S2_2024918 - S2_4433397	34.5	3.9	5.5	-0.3
		LLS_90	D08	A02	S2_2024918 - S2_4433397	31.0	5.4	7.0	-0.5
7.	*qLLS-A03.1*	LLS_70	D08	A03	S3_133697495 - S3_134897269	87.8	13.1	25.4	-0.3
		LLS_90	D08	A03	S3_133697495 - S3_134897269	87.8	33.1	69.0	-1.6
8.	*qRust-A03.1*	Rust_70	D11	A03	S3_133697495 - S3_134897269	87.8	6.7	15.9	0.1
		Rust_80	D11	A03	S3_133697495 - S3_134897269	82.1	16.2	25.0	0.3
		Rust_80	D10	A03	S3_133697495 - S3_134897269	86.8	16.6	35.8	0.4
		Rust_80	D09	A03	S3_133697495 - S3_134897269	87.8	36.6	69.9	1.0
		Rust_80	D07E1	A03	S3_133697495 - S3_134897269	87.8	36.7	59.8	0.6
		Rust_80	D08	A03	S3_133697495 - S3_134897269	87.8	46.3	69.5	0.9
		Rust_80	D06	A03	S3_133697495 - S3_134897269	87.8	53.5	80.8	1.4
		Rust_80	D07E2	A03	S3_133697495 - S3_134897269	87.8	68.3	87.1	2.1
		Rust_90	D09	A03	S3_133697495 - S3_134897269	86.8	23.6	47.4	1.0
		Rust_90	D10	A03	S3_133697495 - S3_134897269	86.8	33.2	63.8	1.0
		Rust_90	D11	A03	S3_133697495 - S3_134897269	86.8	35.2	66.0	1.0
		Rust_90	D07E1	A03	S3_133697495 - S3_134897269	87.8	39.8	66.5	0.7
		Rust_90	D06	A03	S3_133697495 - S3_134897269	87.8	48.0	80.3	1.5
		Rust_90	D07E2	A03	S3_133697495 - S3_134897269	87.8	61.1	83.9	2.0
		Rust_90	D08	A03	S3_133697495 - S3_134897269	87.8	71.6	87.1	2.0
9.	*qLLS-B02.1*	LLS_90	D09	B02	S12_727795 - S12_727845	9.1	11.1	17.6	0.7
10.	*qRust-B02.1*	Rust_70	D14	B02	S12_1692764 - S12_1687343	10.5	56.8	80.9	-0.5

Genome-wide QTL discovery revealed 10 main effect QTLs, with LODs ranging from 3.5 to 71.6 and PVEs ranging from 5.1 to 87.1%. Of these 10 QTLs, three were associated with leaf rust resistance, and seven were associated with LLS resistance. For rust resistance, 3 major main effect QTLs were detected, with LODs ranging from 6.7 to 56.8 and PVEs ranging from 15.9 to 80.9%. Similarly, 7 main effect QTLs were identified for LLS resistance, with LODs ranging from 3.5 to 71.6 and PVEs ranging from 5.6 to 87.1%. Furthermore, all three QTLs were identified as major main effect QTLs for leaf rust. However, of the 7 QTLs for LLS resistance, 5 had major effects (Table 1).

Of the 3 major main effect QTLs detected for leaf rust resistance, *qRust-A02.1* was mapped at 1.2 cM (S2_464902 - S2_768713) on A02, with a LOD value of 11.3 and a PVE of 30.6%, showing a negative additive effect, indicating that a high level of resistance was contributed by GPBD 4. Similarly, a major main effect QTL, *qRust-A03.1*, was mapped on chromosome A03 at 87.8 cM (S3_133697495-S3_134897269), with LODs of 6.7 and 15.9 PVE%. The third major QTL for rust resistance, *qRust-B02.1* (S12_1692764-S12_1687343), was mapped at 10.5 cM with an LOD of 56.8 and 80.9% PVE, and a negative additive effect indicated a high level of the rust-resistant segment from the donor GPBD 4. Although the major QTL detected in B02 for rust resistance was not stable, its high PVE and LOD indicate the necessity for further investigation using a larger genetic population.

In the case of late leaf spot resistance, of the 5 major main effect QTLs detected for LLS resistance, 4 QTLs were mapped to the 5 cM genomic region at the start of chromosome A02. Of these four major main effect QTLs mapped on chromosome A02, *qLLS-A02.1* was mapped at 0.7 cM (S2_880921 - S2_99621) with (14.6–18.3 LOD and 23.3–30.1% PVE), *qLLS-A02.2* was mapped at 2.0 cM (S2_976663 - S2_454537) with (11.7–30.0 LOD to 16.3% PVE), *qLLS-A02.3* was mapped at 5.7 cM (S2_1228446 - S2_1281741) with (9.3–14.0 LOD and 17.3–23.9% PVE), and *qLLS-A02.4* was mapped at 0.1 cM (S2_1240859 - S2_1209628) with (8.7–12.4 LOD and 14.7–18.2% PVE). One main effect QTL on chromosome A02 at 40.5 cM (S2_2024918-S2_4433397) was consistent across the four environments for LLS (Fig. 1B; Table 1). A major main effect QTL, *qLLS-A03.1*, on chromosome A03 was mapped at 87.8 cM (S3_133697495-S3_134897269), with 13.1–33.1 LOD and 25.4–69.0% PVE. The major main effect QTLs located on A02 spanning a physical map distance of 2.08 Mb (0.7–2.8 Mb) were the major QTLs for both rust and LLS, with up to 30.6 and 44.2% PVE, respectively. However, a common genomic region was identified on chromosome A03 for both rust and LLS resistance, with up to 87.1% and 69.0% PVE, respectively (Fig. 1C).

Initially, QTL-seq analyses based on WGRS data for six samples were performed using available genome assemblies for diploid progenitors [24]. Later, the analyses were repeated using a cultivated tetraploid genome assembly for the subspecies *fastigiata* [22]. The reference-guided assembly for the resistant parent GPBD 4 was developed with diploid progenitors and a cultivated tetraploid genome. We observed that the mapping percentage of the tetraploid reference genome was ∼10% greater than that of the diploid progenitor genome in the resistant parent, GPBD 4 (85.07% and 96.58%), Rust_Rbulk (85.24% and 96.68%), Rust_Sbulk (85.34% and 96.77%), LLS_Rbulk (85.01% and 96.34%) and LLS_Sbulk (84.97% and 96.78%). However, there was no significant difference in average mapping depth between the diploid and tetraploid reference genomes, as shown in Supplementary Table 1.

The initial QTL-Seq analysis of the diploid genome-based GPBD 4 assembly revealed a significant colocalized genomic region on chromosome A03 for leaf rust (131.60-134.66 Mb) and LLS resistance (131.67-134.65 Mb) [24]. After analyzing the resistant and susceptible bulks from the QTL region identified in A03, a total of 66 SNPs were effective SNPs identified at a read depth of ≥ 7 for leaf rust and LLS resistance, respectively (Supplementary Table 1). Similarly, QTL-seq analysis of the cultivated tetraploid genome-based GPBD 4 assembly also revealed a colocalised genomic region on the pseudomolecule Chr13 for rust (140.405–144.882 Mb) and LLS (140.808–144.705 Mb) resistance [22]. A total of 3,270 SNPs and 1,620 effective SNPs were identified at a read depth of ≥ 7 for leaf rust and LLS resistance, respectively, after analysing the resistant and susceptible bulks from the QTL region identified on Chr13 (Supplementary Table 1). It is important to note that the source for leaf rust and LLS resistance in GPBD 4 was traced back to *A. cardenasii* while performing analysis with the diploid genome; in contrast, mapping to Chr13 with the tetraploid genome indicated possible translocation of the genomic region controlling resistance from Chr03 to Chr13 after tetraploidization or due to high sequence similarity between the two sub-genomes. This colocalized genomic region yielded more effective SNPs for both diseases in the tetraploid reference genome than in the diploid reference genome.

### Candidate genes for resistance to leaf rust and LLS

**Fig. 2:**
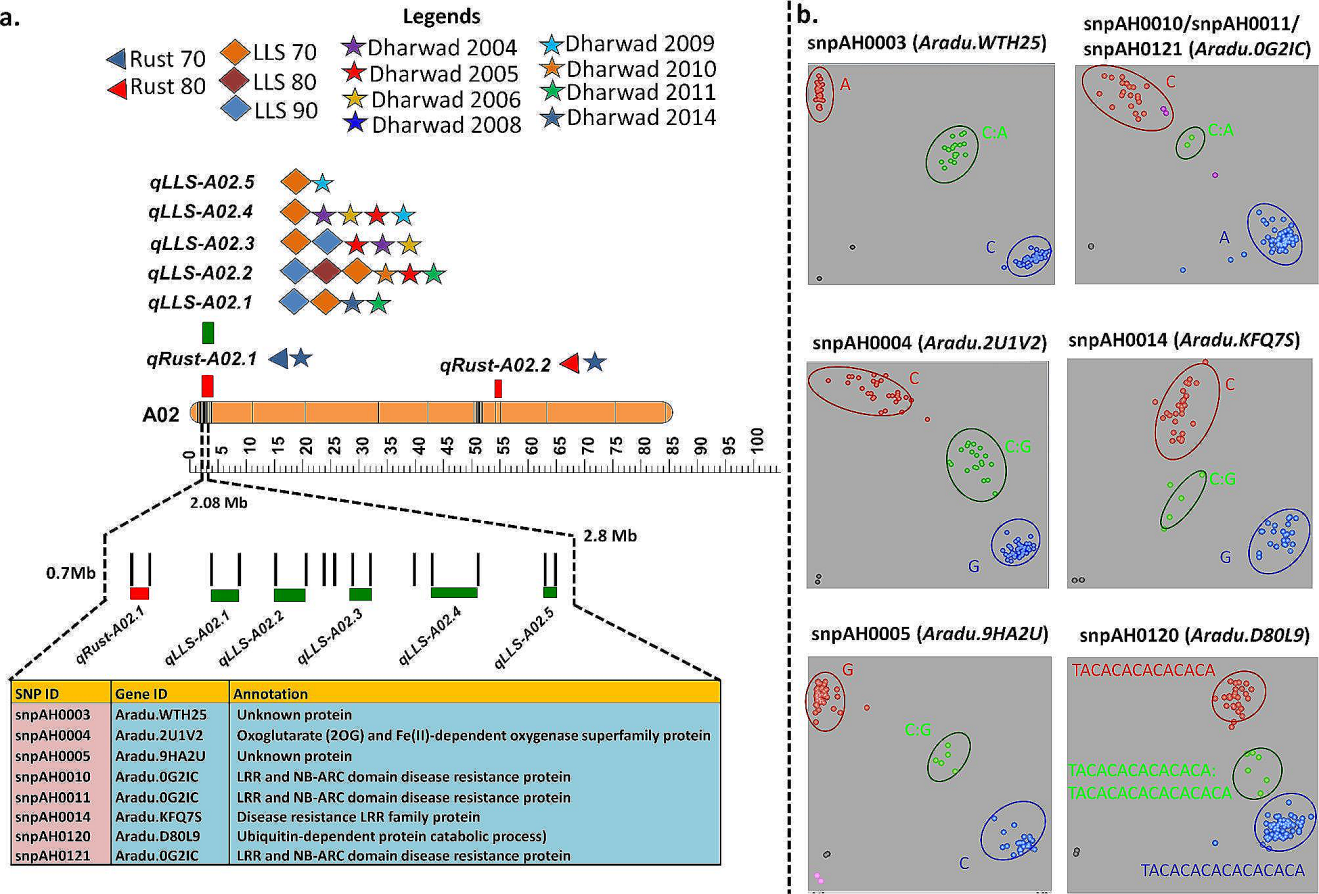
Fine mapping, gene discovery (*LLSR1* gene for LLS resistance), marker development and validation of major main-effect QTLs controlling LLS resistance. (**a**) The 2.08 Mb genomic region on chromosome A02 wherein QTLs were detected across different seasons, with eight candidate resistance genes targeted for marker development. The gene IDs for these candidate resistance genes and KASP assay information are also provided. (**b**) Scatter plot showing the validation results for selected KASP assays (snpAH0003, snpAH0004, snpAH0005, snpAH0003, snpAH0010, snpAH0011, snpAH0014, snpAH0120, and snpAH0121). There are three clusters on each scatter plot. The red cluster indicates the resistant lines, the blue cluster indicates the susceptible lines, and the green clusters are the heterozygotes derived from the crosses between the resistant and susceptible lines

In GBS-based QTL analysis, major main effect QTLs for leaf rust and late leaf spot were identified on chromosomes A02 and A03. The genomic region of length 2.08 Mb (0.7–2.8 Mb) on chromosome A02 comprised 6 adjacent QTL regions (1 for rust resistance and 5 for LLS resistance) detected in various environments (Fig. 2a). Within this region, a total of 245 genes were located (www.peanutbase.org). Of these, 38 candidate genes were found to be affected by 72 GBS-SNPs. The 38 candidate genes included important disease resistance genes, such as LRR and NB-ARC domain disease resistance protein, calmodulin-binding heat-shock protein (*Aradu. MS73B*), DEAD-box ATP-dependent RNA helicase (*Aradu. JB556*), disease resistance LRR family protein (*Aradu. KFQ7S*), ubiquitin-protein ligase (*Aradu. MMY8Q*) and a few unknown proteins (Fig. 2b).

Interestingly, multiple SNPs were identified in the genomic regions of a few candidate genes. For instance, 3 SNPs were identified in the region of the disease resistance gene LRR and NB-ARC domain disease resistance protein (*Aradu.0G2IC*), one SNP (snpAH0014) in the disease resistance LRR family protein (*Aradu. KFQ7S*) (Fig. 2b).

**Fig. 3:**
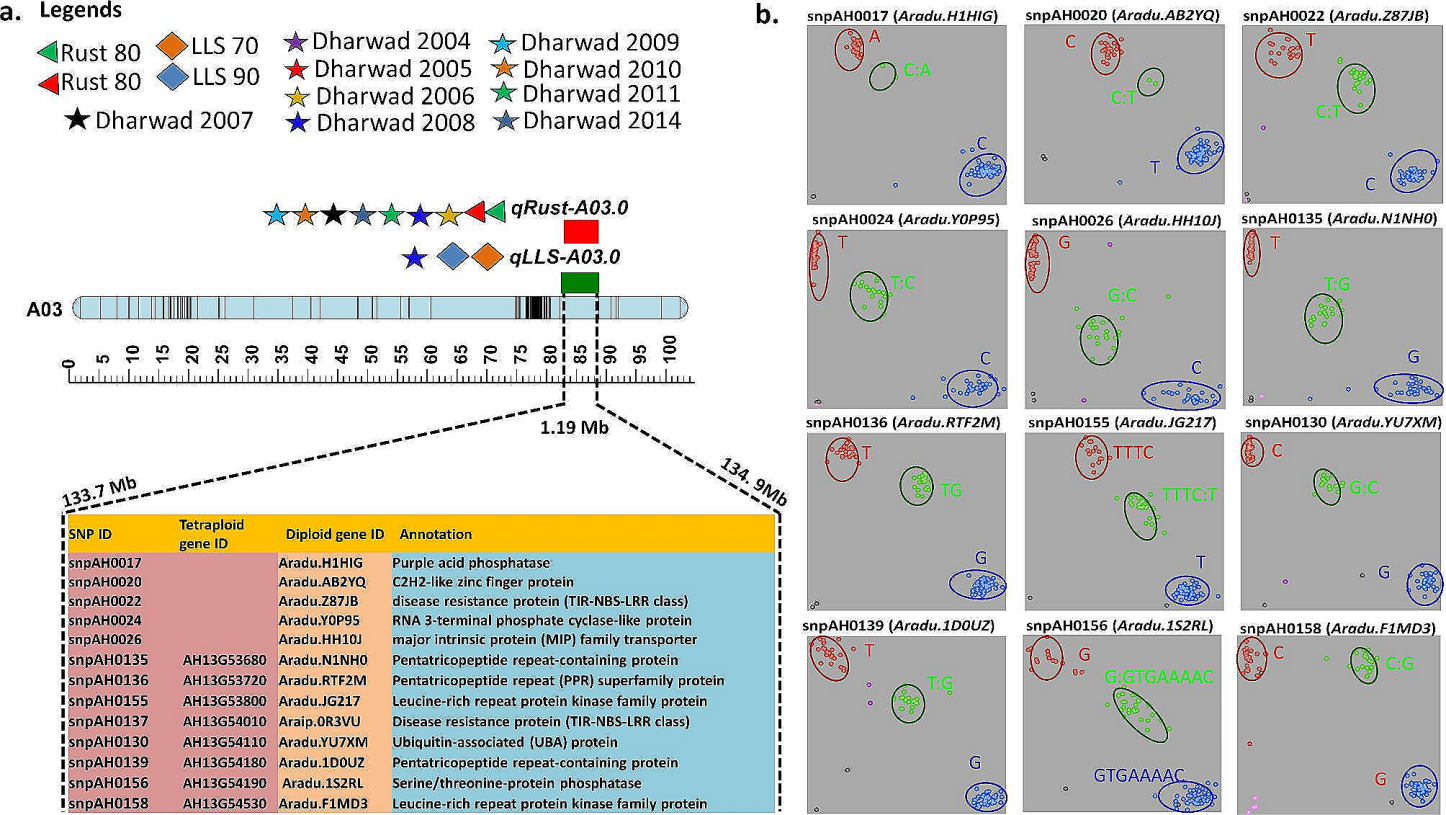
Fine mapping, gene discovery (*LLSR1* gene for LLS resistance and *LR1* gene for LR resistance), marker development and validation for major main-effect colocalised QTLs for rust (*qRust-A03.0) and LLS (qLLS-A03.0)* resistance. Figure (**a**) shows the 1.19 Mb genomic region on chromosome A03, wherein QTLs were detected across different seasons and 13 candidate resistance genes were targeted for marker development. The gene IDs for these candidate resistance genes and KASP assay information are also provided. (**b**) Scatter plot showing the validation results for selected KASP assays (snpAH0017, snpAH0020, snpAH0022, snpAH0024, snpAH0026, snpAH0135, snpAH0136, snpAH0155, snpAH0130, snpAH0139, snpAH0156, and snpAH0158). There are three clusters on each scatter plot. The red cluster indicates the resistant lines, the blue cluster indicates the susceptible lines, and the green clusters are the heterozygotes derived from the crosses between the resistant and susceptible lines

In GBS-based genetic mapping, a common genomic region of 1.19 Mb (133.7-134.9 Mb) on A03 was identified, which overlapped with the regions identified via QTL-seq on chromosome A03 and Chr13 in diploid and tetraploid genomes, respectively. In the QTL-seq analysis, 3,139 SNPs, including 30 nonsynonymous SNPs affecting 25 candidate genes related to plant growth and defense, were detected in the genomic region of 3.06 Mb (131.60-134.66 Mb) on A03 identified for rust resistance using the diploid genome (Fig. 3a).

Similarly, although the genomic region of 2.98 Mb (131.67-134.65 Mb) on A03 was identified for rust resistance using a diploid genome that had 66 SNPs, none were nonsynonymous. Upon repeating the QTL-seq analysis using the tetraploid reference genome, this colocalized genomic region possessed 216 and 171 candidate genes for leaf rust and LLS resistance, respectively. The colocalized genomic region on pseudomolecule Chr13 for rust (140.405–144.882 Mb) and LLS (140.808–144.705 Mb) resistance had 121 and 51 nonsynonymous SNPs, respectively. The number of candidate genes detected through analysis of the tetraploid reference genome was much greater than that detected through analysis of the diploid reference genome. These genes included TIR-NBS-LRR, PPR proteins (*AH13G54010*), C2H2-like zinc finger protein (*Aradu. AB2YQ*), the disease resistance protein TIR-NBS-LRR class (*Aradu. Z87JB, AH13G54010*), pentatricopeptide repeats (*AH13G53720*), leucine-rich repeat kinase family protein (*AH13G53800*), serine/threonine-protein kinase (*AH13G52650*), RNA 3-terminal phosphate cyclase-like protein (*Aradu. HH10J*), C2H2-like zinc finger protein (*Aradu. AB2YQ*), and ubiquitin-associated (UBA)/TS-N domain-containing (*AH13G54110*) (Fig. 3b).

### Development, validation and deployment of genotyping assays for foliar fungal diseases and high oleic acid

From the genomic regions identified via GBS-based genetic mapping and QTL-seq analysis [13, 22], 120 primer pairs were designed and synthesized. Among these primer pairs, 90 showed precise amplification, and 80 exhibited polymorphisms between contrasting genotypes for foliar disease resistance and oleic acid content. Further validation was performed on a panel of diverse genotypes comprising both susceptible and resistant genotypes. The susceptible genotypes included GJ 9, GJ 20, GJGHPS 1, SunOleic 95R, ICGV 07368, ICGV 06420, TMV 2, DH 86, TAG 24, TG 26, ICGV 91114 and JL 24, and the resistant genotypes included GPBD 4 and 11 introgression lines in the genetic background of ICGV 91114, JL 24 and TAG 24 developed through a marker-assisted backcrossing (MABC) approach.

**Fig. 4:**
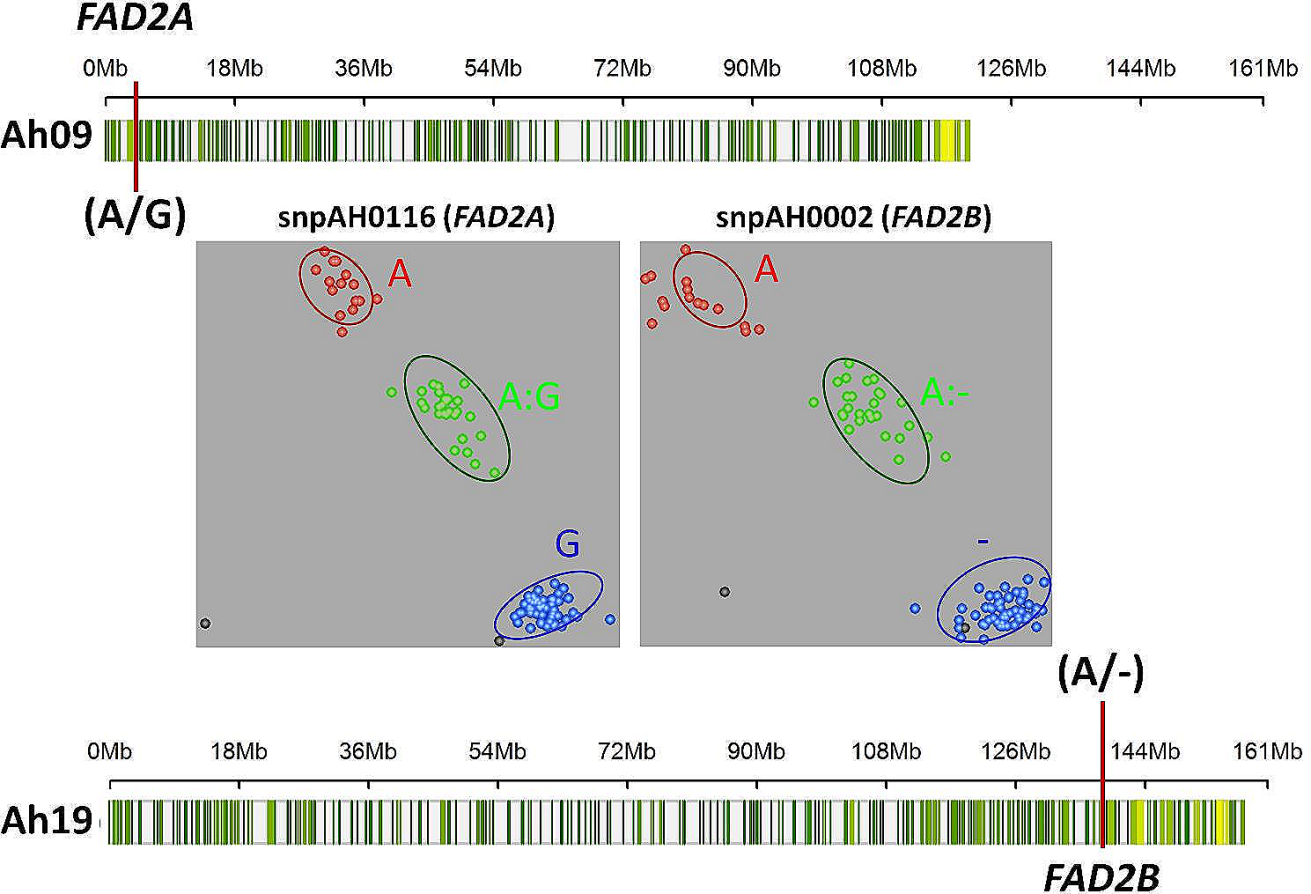
Development and validation of KASP markers for fatty acid desaturase genes controlling high oleic acid. The figure shows the genomic position of *FAD2A* on chromosome 9 and *FAD2B* on chromosome 19 as well as the two validated KASP assays, namely, snpAH0116 (*FAD2A*) and snpAH0002 (*FAD2B*), for high oleic acid. There are three clusters on each scatter plot. The red cluster indicates the resistant lines, the blue cluster indicates the susceptible lines, and the green clusters are the heterozygotes derived from the crosses between the resistant and susceptible lines

In parallel, the KASP assay was designed for 49 SNPs, for which primer validation was successful among the parental genotypes of the RIL population (Table 2; Supplementary Table 2). These KASP markers were validated on a panel of 96 genotypes, which included introgression lines carrying resistance from GPBD 4. Of the KASP markers tested, the eight best performing KASP markers were finalized for further use in breeding. In addition to rust and LLS, we also developed a KASP assay in which the *FAD2A* and *FAD2B* mutant alleles regulate the oleic acid trait (Fig. 4).

**Table 2:** Major QTLs targeted for candidate gene discovery and KASP assay development for late leaf spot (LLS), leaf rust and high oleic acid content

Trait	Gene/Marker interval	Position (cM)	LOD	PVE (%)	Additive effect	KASP assays
**LLS70, LLS80, LLS90**	S2_976663 - S2_454537	2.01	11.69–29.97	16.3–44.2	(-0.584) to (-0.498)	snpAH0010, snpAH0011, snpAH0120, snpAH0121, snpAH0122, snpAH0123, snpAH0126
**LLS70, LLS90**	S2_1228446 - S2_1281741	3.71	9.3-13.97	17.3–23.9	(-0.537) to (-0.664)	snpAH0004, snpAH0005
**LLS70**	S2_2808680 - S2_2024918	23.01	6.53	14.1	-0.4261	snpAH0014
**LLS70, LLS90**	S3_133697495 - S3_134897269	87.81	6.74–68.28	9.8–87.1	(-0.980) to 2.080	snpAH0015, snpAH0016, snpAH0017, snpAH0018, snpAH0020, snpAH0021, snpAH0022, snpAH0024, snpAH0026, snpAH0127, snpAH0128, snpAH0129, snpAH0130, snpAH0131, snpAH0132, snpAH0133, snpAH0134, snpAH0135, snpAH0136, snpAH0137, snpAH0138, snpAH0139, snpAH0140, snpAH0141
**Rust70**	S2_464902 - S2_768713	1.21	11.31	30.6	-0.4861	snpAH0003
**Rust70, Rust80, Rust90**	S3_133697495 - S3_134897269	87.81	6.74–71.56	15.9–87.1	0.12–2.08	snpAH0149, snpAH0150, snpAH0151, snpAH0152, snpAH0153, snpAH0154, snpAH0155, snpAH0156, snpAH0157, snpAH0158, snpAH0159, snpAH0160
**High Oleic acid content**	*ahFAD2A*	65.3	7.32	8.40	-8.70	snpAH0116, snpAH0117
**High Oleic acid content**	*ahFAD2B*	90.5	13.09	25.54	-9.76	snpAH0002

Rust70: Disease score observation for rust at 70 days after sowing; Rust80: Disease score observation for rust at 80 days after sowing; Rust90: Disease score observation for rust at 90 days after sowing; Rust70: Disease score observation for LLS at 70 days after sowing; LLS80: Disease score observation for LLS at 80 days after sowing; LLS90: Disease score observation for LLS at 90 days after sowing

The KASP marker successfully distinguished the *FAD2B* mutant allele on the HTPG platform, while the KASP marker for the *FAD2A* mutant allele failed to perform as expected. Therefore, we sequenced two newly developed high-oleic acid varieties (Girnar 4 and Girnar 5) and completed primer development and validation after sequence analysis of these FAD genes from Girnar 4 and Girnar 5. Finally, we developed a high-throughput genotyping assay platform that is easily accessible through genotyping platforms in India, Sweden and Australia. The transition from genotyping with low-throughput allele-specific markers to high-throughput KASP genotyping has significantly reduced the cost of selection for all three traits to a mere 2.5 USD from 13 USD, bringing more precision and time savings in performing selection and decision making. Encouragingly, KASP assays are now being used regularly in several breeding programs across different countries, including India, Malawi, Mali, Burkina Faso, Nigeria and Uganda.

## Discussion

Resistance to foliar fungal diseases such as rust and late leaf spot are the preferred traits of farmers because of their devastating nature, while a high oleic acid content is an industry- and consumer-preferred trait due to increased shelf life and health benefits. Current groundnut breeding programs have identified resistance to foliar fungal diseases (LLS & rust) and high oleic acid as must have traits. Therefore, in this study, we combined KASP markers for foliar diseases and a high oleic acid content. Leaf rust and late leaf spot are two devastating foliar fungal diseases of groundnuts that cause significant yield loss in all groundnut-growing areas worldwide [23, 34]. Potential resistant sources derived from *Arachis cardenasii*, such as GPBD4 in India and SPT06-06 (GP-NC WS16) in the USA, have been extensively used to improve late leaf spot resistance through conventional breeding approaches. To further accelerate conventional breeding, the development of genetic markers for the introgression of late leaf spot resistance using marker-assisted backcrossing (MABC) has become a focus of groundnut breeding programs. Linked simple sequence repeat (SSR) markers were successfully used for foreground selection in groundnuts [2, 5, 33, 35]. However, genotyping with SSR markers is laborious and time consuming, limiting its utility in molecular breeding. Advances in sequencing and genotyping technologies resulted in the discovery of new genotyping platforms. For instance, diagnostic KASP markers have proven to be a rapid genotyping platform for foreground selection, thereby accelerating marker-assisted introgressions. In this study, we reported the development and scaling up of newly developed and validated high-quality KASP markers for high oleic acid content, leaf rust and late leaf spot.

In groundnut, QTL-seq has been successfully used for the discovery of genomic regions and candidate genes associated with LLS and rust resistance. To identify the genomic regions for LLS and rust resistance, diploid reference genomes (*A. ipaensis* and *A. duranensis*) were initially used [13], and the genomic region on chromosome A03 was identified; subsequently, cultivated tetraploid reference genomes (Shitoqui) were used [22], and the genomic region on chromosome B03 (chr13) in the tetraploid genome was identified. Furthermore, to confirm the genomic regions identified in QTL-Seq studies, we used a genotyping-by-sequencing (GBS)-based genetic mapping approach to identify the QTLs linked with late leaf spot and rust resistance. The overlapping genomic regions from QTL-Seq studies and the QTLs identified via GBS-based genetic mapping were used for the development of KASP markers. A total of 47 SNPs with a Δ-SNP index of -1 from both studies were used for the development of KASP markers for LLS and rust. Among these 47 SNPs, 39 SNPs were validated on a panel of resistant and susceptible lines for LLS and rust, and 2 KASPs for high oleic acid from the A- and B-genomes were validated successfully on contrasting oleic acid-containing lines.

All the targeted SNPs were selected from the genomic regions of potential disease resistance genes reported earlier in various crop plants. According to the pedigrees of the resistance donor and reference genomes used for analysis, the resistance QTLs for rust and LLS resistance have been reported either on chromosomes A02 or B02 or on chromosomes A03 or B03 of both sub-genomes in various studies. For instance, a common region was identified for rust and LLS resistance on chromosome A03 by [24] using a QTL-seq approach with diploid reference genomes. Using the same dataset, QTL-Seq analysis of the tetraploid genome was used to identify the region on chromosome chr13 [22]. There are also reports on the identification of resistant QTLs on chromosome A05 in the Tifrunner genetic background using the QTL-Seq approach [18]. However, in this study, potential markers for rust and LLS were developed and successfully validated for chromosome A03 [24] and chr13 (B03; [22]). Interestingly, in this study, which used GBS-based genetic mapping, an additional major QTL (*qRust-B02.1*) identified on chromosome B02 for rust resistance with a PVE of 80.9% is also a promising genomic region because of its high phenotypic variance. In the future, this region can be further explored using fine mapping to identify potential resistance alleles for rust and LLS resistance.

**Fig. 5:**
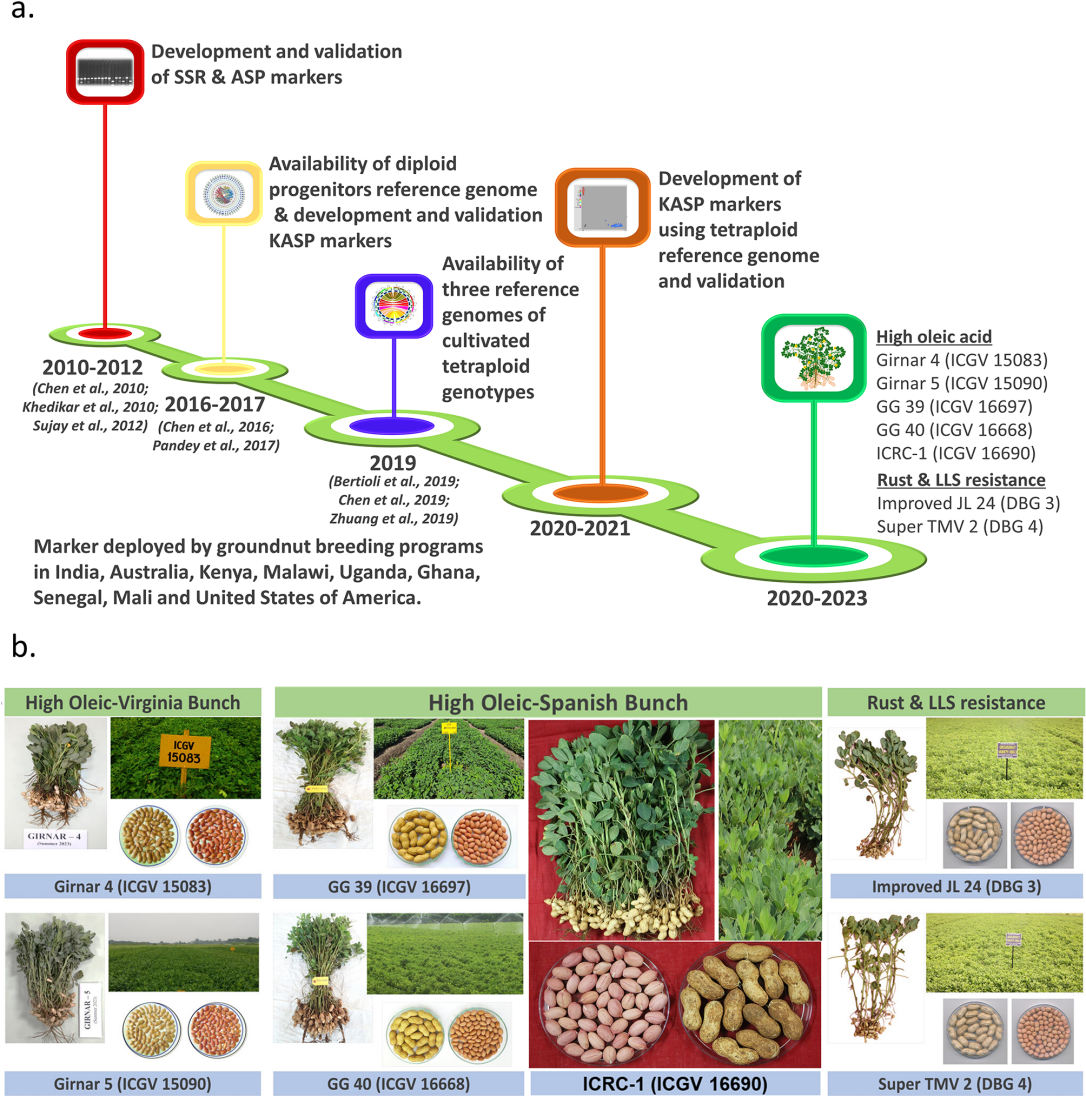
Lab-to-field applications and impact of KASP markers in groundnuts. (**a**) Illustration of the roadmap for developing diagnostic KASP markers; (**b**) Molecular breeding products using diagnostic markers for foliar fungal diseases and a high oleic acid content

Fatty acid desaturase enzyme catalyzes desaturation of oleic to linoleic acid, and it has two copies in both sub-genomes of cultivated groundnut. Here, we developed two markers for high oleic acid content (snpAH0116 from the A sub-genome and snpAH002 from the B sub-genome) located in the genic region of fatty acid desaturase (FAD) from the A and B sub-genomes, respectively (Fig. 5a). Similarly, KASP markers for LLS (snpAH0010, snpAH0011, and snpAH00121) were located in the genic regions of the LRR and NB-ARC domain disease resistance proteins (*Aradu.0G2IC*). KASP snpAH0014 was also developed from a protein/LRR family protein in the disease resistance family (*Aradu.KFQ7S*). snpAH0022 is a disease resistance protein (TIR-NBS-LRR class) (*Aradu.Z87JB*). NBS-LRR proteins recognize effectors secreted by pathogens directly or indirectly that in turn activate downstream signaling pathways, leading to activation of the plant defense response [36]. The KASP marker snpAH0120 represents an important gene, SKP1-like protein (*Aradu.D80L9*). The SKP1 protein is a component of the SKP1 (S-phase kinase-associated protein 1) complex and regulates the ubiquitination of proteins targeted for proteasomal degradation. Ubiquitination is implicated in many cellular processes, including plant defense responses [37]. Moreover, it plays a role in plant hormone signaling [38] and the accumulation of nucleotide-binding leucine-rich repeat-type immune receptors [39]. Induced expression of SKP1 was observed in *N. benthamiana* after infection with potato virus X, which is an indication of the involvement of ubiquitination in plant defense [40], and the upregulation of Arabidopsis SKP1 (ASK1 and ASK2) was shown to be required for successful Agrobacterium-mediated plant transformation [41]. snpAH0004 is located in the genic region of oxoglutarate (2OG) and Fe(II)-dependent oxygenase superfamily protein *Aradu.2U1V2*. 2OGs are reportedly involved in melatonin metabolism and subsequently affect plant responses to cold, heat, salt, drought, heavy metal stress, and pathogen invasion [42]. SnpAH0017 was developed from the genic region of purple acid phosphatase (*Aradu.H1HIG*). It has been reported that an optimal level of PAP maintains resistance against *Pseudomonas syringae*. It has also been reported that a mutation in PAP results in susceptibility to *Pseudomonas syringae* [43]. SnpAH0018 was located in the genic region of the P-loop containing nucleoside triphosphate hydrolase (*Aradu.14 × 1M*). The P-loop-containing nucleotide triphosphate hydrolase superfamily contains several kinases that are known to be involved in disease resistance [44]. C2H2-like zinc finger proteins positively modulate the expression levels of stress-related genes by directly binding to TACAAT motifs in the promoter regions of pathogen-related genes such as *ENHANCED DISEASE SUSCEPTIBILITY1*, *PHYTOALEXIN DEFICIENT4*, and *PATHOGENESIS-RELATED GENE1* (*PR1*, *PR2*, and *PR5*) [45]. KASP was developed from the transcription factor TCP2 (*AH13G52920*). TCPs regulate plant development and defense responses by stimulating the biosynthetic pathways of bioactive metabolites, such as brassinosteroid (BR), jasmonic acid (JA) and flavonoids [46]. SnpAH0026 was developed from the major intrinsic protein (MIP) family transporter *Aradu.HH10J*. MIP isoforms are transporters of hydrogen peroxide. H_2_O_2_ experiences cross talk with immune pathways, such as those involved in systemic acquired resistance (SAR) and pathogen-associated molecular pattern-triggered immunity (PTI), to regulate plant disease resistance [47]. Topless-related protein 1 (*AH13G53060*) was also targeted for the development of the KASP marker snpAH0151. TOPLESS (TPL)/TOPLESS-RELATED (TPR), TPL has been linked to plant stress responses through several phytohormones [48] and is also involved in plant responses to pathogen attack. Arabidopsis plants with depleted TPL/TPR activity were reported to be more susceptible to infection due to disruption of SNC1-mediated immune responses [49]. Additionally, TPL is recruited by TGA family bZIP transcription factors that control development and stress responses via ROXY adaptor proteins to repress TGA targets [50]. SnpAH0159 was targeted from the genic region of CBL-interacting serine/threonine-protein kinase (*AH13G54550*). Calcineurin B-like proteins (CBLs) are a family of calcium sensor proteins that interact with a group of serine/threonine kinases designated CBL-interacting protein kinases (CIPKs). CBL-CIPK complexes are crucially involved in relaying plant responses to many environmental signals and in regulating ion fluxes [51] (Table 3).

**Table 3:** Details of validated KASP assays for resistance to leaf rust and LLS and high oleic acid content tables

QTLs	KASP ID	SNP	Chromosome	Genomic Position (bp)	Trait	Gene ID identified at the time of analysis	Shitoqui V2 gene ID	Tifrunner V1/V2 gene IDs	Annotation
*qLLS-A02.1*	snpAH0010	C/A	A02	435,829	LLS	Aradu.0G2IC	AH02G00200	*Arahy.B6JPF5*	LRR - NB-ARC disease resistance protein
	snpAH0011	C/T	A02	436,779	LLS	Aradu.0G2IC	AH02G00200	*Arahy.B6JPF5*	LRR - NB-ARC disease resistance protein
	snpAH0120	TACACACACACACACA**/**TACACACACACACA	A02	260,156	LLS	Aradu.D80L9	AH02G03390	*Arahy.LDA2L2*	SKP1-like protein
	snpAH0121	G/C	A02	442,214	LLS	Aradu.0G2IC	AH02G00200	*Arahy.B6JPF5*	LRR - NB-ARC disease resistance protein
*qLLS-A02.2*	snpAH0004	C/G	A02	1,271,582	LLS	Aradu.2U1V2	AH02G01030	*Arahy.KAP8H4*	Oxoglutarate (2OG and Fe-II-dependent oxygenase superfamily protein
	snpAH0005	G/C	A02	1,156,975	LLS	Aradu.9HA2U	AH02G00920	*Arahy.GIK8C1*	unknown protein
*qLLS-A02.3*	snpAH0014	C/G	A02	1,932,131	LLS	Aradu.KFQ7S	AH02G01460	*Arahy.IKT7HX*	disease resistance family protein/LRR family protein
*qLLS-A03.1*	snpAH0015	T/A	A03	133,497,786	Rust	Aradu.6U7NW	AH13G52070	*Arahy.JT7ZMS*	uncharacterized protein
	snpAH0016	C/T	A03	131,788,843	Rust	Aradu.PNQ8T	AH17G26960	*0*	Unknown protein
	snpAH0017	A/C	A03	131,739,517	Rust	Aradu.H1HIG	AH03G47990	*Arahy.06GG2J*	Purple acid phosphatase
	snpAH0018	G/A	A03	131,937,796	Rust	Aradu.14 × 1 M	AH03G48170	*Arahy.VTXV28*	P-loop containing nucleoside triphosphate hydrolase
	snpAH0020	C/T	A03	132,022,031	Rust	Aradu.AB2YQ	AH13G50920	*Arahy.T3TBG0*	C2H2-like zinc finger protein
	snpAH0021	C/G	A03	133,527,661	Rust	Aradu.KU7EH	AH13G52090	*Arahy.GQD7YX*	UDP-Glycosyltransferase superfamily protein
	snpAH0022	T/C	A03	133,780,314	Rust	Aradu.Z87JB	AH13G52390	*Arahy.R8KUIR*	Disease resistance protein (TIR-NBS-LRR class
	snpAH0024	T/C	A03	134,225,429	Rust	Aradu.Y0P95	AH13G52930	*Arahy.3N5Q80*	RNA 3-terminal phosphate cyclase-like protein
	snpAH0026	G/C	A03	134,613,173	Rust	Aradu.HH10J	AH13G53880	*Arahy.L5GP6Q*	Major intrinsic protein (MIP family transporter)
	snpAH0128	C/T	Chr13	142,435,734	LLS	AH13G52890	AH13G51110	*Arahy.QFR843*	Receptor like protein 29
	snpAH0129	AG/A	Chr13	143,296,024	LLS	AH13G53530	0	*0*	protein coding
	snpAH0130	C/G	Chr13	143,975,755	LLS	AH13G54110	AH13G52470	*Arahy.AI5VH5*	Ubiquitin-associated (UBA/protein)
	snpAH0132	T/TAA	Chr13	141,981,452	LLS + Rust	AH13G52650	AH13G50800	*Arahy.AL67ST*	Probable serine/threonine-protein kinase
	snpAH0133	TGTGCAGCATCCTAAAGAATTCAGGC/T	Chr13	141,990,549	LLS + Rust	AH13G52670	AH13G50820	*Arahy.0A9D4I*	Kinesin-like protein NACK1
	snpAH0134	CTT/C	Chr13	142,829,832	LLS + Rust	AH13G53120	AH03G48830	*Arahy.C3CR7W*	Serine/threonine protein phosphatase
	snpAH0135	T/G	Chr13	143,462,288	LLS + Rust	AH13G53680	AH13G52000	*Arahy.VTBJ7F*	Pentatricopeptide repeat-containing protein
	snpAH0136	C/T	Chr13	143,504,769	LLS + Rust	AH13G53720	AH13G52030	*Arahy.SAUI5N*	Pentatricopeptide repeat (PPR superfamily protein)
	snpAH0137	C/G	Chr13	143,852,868	LLS + Rust	AH13G54010	AH13G55880	*Arahy.F01FM8*	Disease resistance protein (TIR-NBS-LRR class
	snpAH0138	T/G	Chr13	143,922,005	LLS + Rust	AH13G54070	AH13G52410	*Arahy.ZGTC82*	Histidine kinase
	snpAH0140	G/T	Chr13	144,783,964	LLS + Rust	AH13G54550	AH13G53400	*Arahy.G6JZIF*	CBL-interacting serine/threonine-protein kinase 12
	snpAH0141	A/G	Chr13	144,789,658	LLS + Rust	AH13G54850	AH13G53840	*Arahy.JR5BIL*	MACPF domain-containing protein CAD1
*qRust-A02.1*	snpAH0003	A/C	A02	768,480	LLS	Aradu.WTH25	AH02G00490	*Arahy.02:67ZQRX*	unknown protein
	snpAH0149	A/AT	Chr13	142,468,300	Rust	AH13G52920	AH13G51140	*Arahy.JF9204*	Transcription factor TCP2
*qRust-A03.1*	snpAH0150	CT/T	Chr13	142,583,511	Rust	AH13G52970	AH13G51650	*Arahy.3VLA0M*	RING/U-box superfamily protein
	snpAH0151	G/GA	Chr13	142,719,262	Rust	AH13G53060	AH13G51330	*Arahy.GHW83A*	Topless-related protein 1
	snpAH0152	A/AGAGTAACC	Chr13	143,050,728	Rust	AH13G53300	AH13G13480	*Arahy.QP8I27*	Protein kinase superfamily protein
	snpAH0153	AGT/A	Chr13	143,320,048	Rust	AH13G53550	AH13G51840	*Arahy.8GYA0C*	F-box only protein 6
	snpAH0154	T/TAG	Chr13	143,411,025	Rust	AH13G53630	AH13G51920	*Arahy.H5PMU0*	MACPF domain-containing protein At1g14780
	snpAH0155	TTTC/T	Chr13	143,607,528	Rust	AH13G53800	AH13G52160	*Arahy.BKZ08E*	Leucine-rich repeat protein kinase family protein
	snpAH0156	G/GTGAAAAC	Chr13	144,046,233	Rust	AH13G54190	AH13G13290	*Arahy.909AJ0*	Serine/threonine-protein phosphatase
	snpAH0159	GA/G	Chr13	144,452,692	Rust	AH13G54550	AH13G53400	*Arahy.G6JZIF*	CBL-interacting serine/threonine-protein kinase 12
	snpAH0160	C/CTATA	Chr13	144,798,806	Rust	AH13G54850	AH13G53840	*Arahy.JR5BIL*	MACPF domain-containing protein CAD1
*qOLE-Ah09.1*	snpAH0116	A/G	Ah09	1,224,425	HOA	FAD2A	AH09G29670		Fatty acid desaturase from A subgenome
*qOLE-Ah19.1*	snpAH0002	A/-:-	Ah19	B-genome	HOA	FAD2B	AH19G38370		Fatty acid desaturase from B subgenome

These newly developed KASP markers were made publicly available (https://excellenceinbreeding.org/module3/kasp) before this publication, with genotyping options available in India, Sweden and Australia. To date, these markers have been successfully used in breeding programs in multiple countries, such as India, Australia, Kenya, Malawi, Uganda, Ghana, Senegal, Mali and the United States. The deployment of low-throughput allele-specific markers and these newly developed high-throughput KASP assays has resulted in the development and release of commercial cultivation of five high-oleic acid groundnut varieties, namely, Girnar 4 (ICGV 15083), Girnar 5 (ICGV 15090), GG 39 (ICGV 16697), GG 40 (ICGV 16668) and ICRC-1 (ICGV 16690), in India for cultivation in Gujarat, Rajasthan, Karnataka, Andhra Pradesh and Tamil Nadu. Low-throughput genotyping assays, such as allele-specific and simple sequence repeat (SSR) genotyping, are being used in developing two new foliar disease-resistant varieties, namely, Improved JL 24 (DBG 3) and Super TMV 2 (DBG 4), at the University of Agricultural Sciences (UAS), Dharwad, India. National Agricultural Research Stations (NARSs), such as the Indian Council of Agricultural Research-Directorate of Groundnut Research (ICAR-DGR), Junagadh, India [10]; Acharya N.G. Ranga Agricultural University, Andhra Pradesh, India; Professor Jayashankar Telangana State Agricultural University, Telangana, India [33]; and University of Agricultural Sciences Dharwad, India [6, 12], used allele-specific and KASP markers, leading to the development of improved groundnut varieties with enhanced LLS and rust resistance and oleic acid content. The newly developed diagnostic kit for LLS, rust and oleic acid has 10 KASP markers, including two KASPs for the selection of alleles with high oleic acid content (Fig. 3). These high-throughput genotyping assays are also being developed for other traits, such as fresh seed dormancy [52, 53] and seed weight [54, 55], and for identifying true allotetraploids derived from crosses of the A- and B/K-genomes of Arachis diploid species [56].

## Conclusion

Although the contributions of homologous chromosomes (A02/B02 and A03/B03) from the two sub-genomes are debated, these validated markers provide opportunities for routine deployment in groundnut breeding programs. The reason for this confusion is mostly because of the high sequence similarity between the two sub-genomes, which poses serious challenges to existing software for mapping such associated genomic regions. Nevertheless, the authors still vouch that the contribution of resistance comes from the A sub-genome (A02 and A03) due to the resistance source being *A. cardenasii*, which belongs to the A genome. Most importantly, a diagnostic kit for LLS, rust and high oleic acid content will be an important genomic tool for improving foliar disease resistance and high oleic acid content in groundnuts. Moreover, the successful deployment of genomic resources underpinned a significant milestone in our research, effectively advancing the development and release of improved groundnut varieties (Fig. 5b). Selection in breeding progenies can be performed for four traits (resistance to rust and LLS, high oleic acid content and fresh seed dormancy) using only 10 SNPs, making large-scale deployment most affordable and cost-effective. Recently, we also optimized a protocol to perform single-seed-based genotyping in groundnuts and started processing a large number of samples in groundnut breeding programs for selection even before seeds went to the field [26], thereby saving resources and time. The integration of such technologies is highly important for reducing the duration of breeding cycles.

### Electronic supplementary material

Below is the link to the electronic supplementary material.

Supplementary Material 1

## Data Availability

The data presented in the study are deposited in NCBI, accession number PRJNA999952 and PRJNA992198.

## Abbreviations

LLS: Late Leaf Spot
MEGS: Marker-Based Early Generation Advancement
QTL: Quantitative trait locus
KASP: Kompetitive allele-specific PCR
MABC: Marker-assisted back-cross
SSR: Simple sequence repeat
RIL: Recombinant inbred line population
PVE: Phenotypic variance explained
AICRP-G: All India Coordinated Research Project on Groundnut
DAS: Days after sowing
FAD: Fatty acid desaturase
GBS: Genotyping-by-sequencing
cM: Centi-Morgan
SNP: Single nucleotide polymorphism

## References

[1] FAOSTAT. (2020), http://faostat3.fao.org/home/index.html.

[2] Shasidhar Y, Variath MT, Vishwakarma MK (2020). Improvement of three popular Indian groundnut varieties for foliar disease resistance and high oleic acid using SSR markers and SNP array in marker-assisted backcrossing. Crop J.

[3] Subrahmanyam P, Williams JH, McDonald D, Gibbons RW (1984). The influence of foliar diseases and their control by selective fungicides on a range of groundnut (*Arachis hypogaea* L.) genotypes. Ann Appl Biol.

[4] Pandey MK, Pandey AK, Kumar R (2020). Translational genomics for achieving higher genetic gains in groundnut. Theor Appl Genet.

[5] Varshney RK, Pandey MK, Janila P (2014). Marker-assisted introgression of a QTL region to improve rust resistance in three elite and popular varieties of peanut (*Arachis hypogaea* L). Theor Appl Genet.

[6] Sharanabasappa BY, Bhat RS. (2016) Development of late leaf spot and rust resistant backcross lines in Jl 24 variety of groundnut (*Arachis hypogaea* L.).

[7] Kolekar RM, Sukruth M, Shirasawa K (2017). Marker-assisted backcrossing to develop foliar disease‐resistant genotypes in TMV 2 variety of peanut (*Arachis hypogaea* L). Plant Breed.

[8] Nawade B, Mishra GP, Radhakrishnan T (2019). Development of high oleic peanut lines through marker-assisted introgression of mutant ahFAD2 alleles and its fatty acid profiles under open-field and controlled conditions. 3 Biotech.

[9] Janila P, Pandey MK, Shasidhar Y (2016). Molecular breeding for introgression of fatty acid desaturase mutant alleles (ahFAD2A and ahFAD2B) enhances oil quality in high and low oil containing peanut genotypes. Plant Sci.

[10] Bera SK, Kamdar JH, Kasundra SV (2018). Improving oil quality by altering levels of fatty acids through marker-assisted selection of ahfad2 alleles in peanut (*Arachis hypogaea* L). Euphytica.

[11] Huang B, Qi F, Sun Z et al. (2019) Marker-assisted backcrossing to improve seed oleic acid content in four elite and popular peanut (*Arachis hypogaea* L.) cultivars with high oil content. Breed Sci 18107. 10.1270/jsbbs.18107 PMC6711728 31481832

[12] Jadhav MP, Gangurde SS, Hake AA et al. (2021) Genotyping-by-sequencing based genetic mapping identified major and consistent genomic regions for productivity and quality traits in peanut. Front Plant Sci 2034. 10.3389/fpls.2021.668020 PMC8495222 34630444

[13] Pandey MK, Roorkiwal M, Singh VK (2016). Emerging genomic tools for legume breeding: current status and future prospects. Front Plant Sci.

[14] Varshney RK, Singh VK, Kumar A (2018). Can genomics deliver climate-change ready crops?. Curr Opin Plant Biol.

[15] Bertioli DJ, Cannon SB, Froenicke L (2016). The genome sequences of *Arachis duranensis* and *Arachis Ipaensis*, the diploid ancestors of cultivated peanut. Nat Genet.

[16] Chen X, Li H, Pandey MK (2016). Draft genome of the peanut A-genome progenitor (*Arachis duranensis*) provides insights into geocarpy, oil biosynthesis, and allergens. Proc Natl Acad Sci.

[17] Lu Q, Li H, Hong Y, Zhang G, Wen S, Li X, Zhou G, Li S, Liu H, Liu H, Liu ZJ (2018). Genome sequencing and analysis of the peanut B-genome progenitor (*Arachis Ipaensis*). Front Plant Sci.

[18] Clevenger J, Chu Y, Chavarro C (2017). Genome-wide SNP genotyping resolves signatures of selection and tetrasomic recombination in peanut. Mol Plant.

[19] Pandey MK, Agarwal G, Kale SM (2017). Development and evaluation of a high density genotyping ‘Axiom_Arachis’ array with 58 K SNPs for accelerating genetics and breeding in groundnut. Sci Rep.

[20] Bertioli DJ, Jenkins J, Clevenger J (2019). The genome sequence of segmental allotetraploid peanut *Arachis hypogaea*. Nat Genet.

[21] Chen X, Lu Q, Liu H (2019). Sequencing of cultivated peanut, *Arachis hypogaea*, yields insights into genome evolution and oil improvement. Mol Plant.

[22] Zhuang W, Chen H, Yang M (2019). The genome of cultivated peanut provides insight into legume karyotypes, polyploid evolution and crop domestication. Nat Genet.

[23] Sujay V, Gowda MVC, Pandey MK (2012). QTL analysis and construction of consensus genetic map for foliar diseases resistance based on two RIL populations in cultivated groundnut (*Arachis hypogaea* L). Mol Breed.

[24] Pandey MK, Khan AW, Singh VK (2017). QTL-seq approach identified genomic regions and diagnostic markers for rust and late leaf spot resistance in groundnut (*Arachis hypogaea* L). Plant Biotechnol J.

[25] Shirasawa K, Bhat RS, Khedikar YP (2018). Sequencing analysis of genetic loci for resistance for late leaf spot and rust in peanut (*Arachis hypogaea* L). Front Plant Sci.

[26] Parmar S, Deshmukh DB, Kumar R (2021). Single seed-based high-throughput genotyping and Rapid Generation Advancement for Accelerated Groundnut Genetics and Breeding Research. Agronomy.

[27] Gowda MVC, Motagi BN, Naidu GK et al. (2002) GPBD 4: a Spanish bunch groundnut genotype resistant to rust and late leaf spot.

[28] Bradbury PJ, Zhang Z, Kroon DE (2007). TASSEL: software for association mapping of complex traits in diverse samples. Bioinformatics.

[29] Van Ooijen JW. (2006) JoinMap® 4, Software for the calculation of genetic linkage maps in experimental populations. Kyazma BV, Wageningen 33.

[30] Voorrips R (2002). MapChart: software for the graphical presentation of linkage maps and QTLs. J Hered.

[31] Kosambi DD. (1944) The estimation of map distances from recombination values. DD Kosambi Sel Work Math Stat 125–30.

[32] Wang S, Basten CJ, Zeng ZB. (2007) Windows QTL cartographer 2.5. Department of Statistics, North Carolina State University, Raleigh, NC. http://statgen.ncsu.edu/qtlcart/WQTLCart.htm (accessed 12 Jan. 2023).

[33] Deshmukh DB, Marathi B, Sudini HK (2020). Combining high oleic acid trait and resistance to late leaf spot and rust diseases in groundnut (*Arachis hypogaea* L). Front Genet.

[34] Gangurde SS, Kumar R, Pandey AK et al. (2019) Climate-smart groundnuts for achieving high productivity and improved quality: current status, challenges, and opportunities. Genomic Des Clim Oilseed Crop 133–72.

[35] Chu Y, Clevenger JP, Holbrook CC (2022). Registration of two peanut recombinant inbred lines (TifGP-5 and TifGP‐6) resistant to late leaf spot disease. J Plant Regist.

[36] DeYoung BJ, Innes RW (2006). Plant NBS-LRR proteins in pathogen sensing and host defense. Nat Immunol.

[37] Furlan G, Klinkenberg J, Trujillo M (2012). Regulation of plant immune receptors by ubiquitination. Front Plant Sci.

[38] Santner A, Estelle M (2009). Recent advances and emerging trends in plant hormone signaling. Nature.

[39] Cheng YT, Li Y, Huang S (2011). Stability of plant immune-receptor resistance proteins is controlled by SKP1-CULLIN1-F-box (SCF)-mediated protein degradation. Proc Natl Acad Sci.

[40] Ye C, Chen S, Payton M (2013). TGB p3 triggers the unfolded protein response and SKP 1-dependent programmed cell death. Mol Plant Pathol.

[41] Anand A, Rojas CM, Tang Y, Mysore KS (2012). Several components of SKP1/Cullin/F-box E3 ubiquitin ligase complex and associated factors play a role in Agrobacterium‐mediated plant transformation. New Phytol.

[42] Wei S, Zhang W, Fu R, Zhang Y (2021). Genome-wide characterization of 2-oxoglutarate and fe (II)-dependent dioxygenase family genes in tomato during growth cycle and their roles in metabolism. BMC Genomics.

[43] Ravichandran S, Stone SL, Benkel B (2015). Optimal level of purple acid phosphatase5 is required for maintaining complete resistance to *Pseudomonas syringae*. Front Plant Sci.

[44] Arya P, Acharya V (2018). Plant STAND P-loop NTPases: a current perspective of genome distribution, evolution, and function: plant STAND P-loop NTPases: genomic organization, evolution, and molecular mechanism models contribute broadly to plant pathogen defense. Mol Genet Genomics.

[45] Shi H, Chan Z (2014). The cysteine2/histidine2-type transcription factor ZINC FINGER OF *ARABIDOPSIS THALIANA* 6‐activated C‐REPEAT‐BINDING FACTOR pathway is essential for melatonin‐mediated freezing stress resistance in Arabidopsis. J Pineal Res.

[46] Li S (2015). The *Arabidopsis thaliana* TCP transcription factors: a broadening horizon beyond development. Plant Signal Behav.

[47] Bienert GP, Chaumont F (2014). Aquaporin-facilitated transmembrane diffusion of hydrogen peroxide. Biochim Biophys Acta (BBA)-General Subj.

[48] Causier B, Lloyd J, Stevens L, Davies B (2012). TOPLESS corepressor interactions and their evolutionary conservation in plants. Plant Signal Behav.

[49] Zhu Z, Xu F, Zhang Y (2010). Arabidopsis resistance protein SNC1 activates immune responses through association with a transcriptional corepressor. Proc Natl Acad Sci.

[50] Uhrig JF, Huang L-J, Barghahn S (2017). CC-type glutaredoxins recruit the transcriptional corepressor TOPLESS to TGA-dependent target promoters in *Arabidopsis thaliana*. Biochim Biophys Acta (BBA)-Gene Regul Mech.

[51] Hashimoto K, Eckert C, Anschütz U (2012). Phosphorylation of calcineurin B-like (CBL) calcium sensor proteins by their CBL-interacting protein kinases (CIPKs) is required for full activity of CBL-CIPK complexes toward their target proteins. J Biol Chem.

[52] Kumar R, Janila P, Vishwakarma MK (2020). Whole-genome resequencing‐based QTL‐seq identified candidate genes and molecular markers for fresh seed dormancy in groundnut. Plant Biotechnol J.

[53] Bomireddy D, Gangurde SS, Variath MT (2022). Discovery of major quantitative trait loci and candidate genes for fresh seed dormancy in groundnut. Agronomy.

[54] Gangurde SS, Khan AW, Janila P et al. (2022) Whole-genome sequencing based discovery of candidate genes and diagnostic markers for seed weight in groundnut. Plant Genome e20265. 10.1002/tpg2.20265 PMC12807278 36478184

[55] Gangurde SS, Pasupuleti J, Parmar S (2023). Genetic mapping identifies genomic regions and candidate genes for seed weight and shelling percentage in groundnut. Front Genet.

[56] Levinson CM, Bertioli D, Chu Y (2021). Development and applications of KASP markers distinguishing A-and B/K-genomes of *Arachis*. Euphytica.

