# Correction: High-throughput diagnostic markers for foliar fungal disease resistance and high oleic acid content in groundnut

**DOI:** 10.1186/s12870-024-05039-y

**Published:** 2024-04-25

**Authors:** Manish K. Pandey, Sunil S. Gangurde, Yaduru Shasidhar, Vinay Sharma, Sandip M. Kale, Aamir W. Khan, Priya Shah, Pushpesh Joshi, Ramesh S. Bhat, Pasupuleti Janila, Sandip K. Bera, Rajeev K. Varshney

**Affiliations:** 1 International Crops Research Institute for the Semi-Arid Tropics (ICRISAT), Hyderabad, India; 2 University of Agricultural Sciences, Dharwad, India; 3 ICAR-Directorate of Groundnut Research, Junagadh, India; 4 Centre for Crop and Food Innovation, WA State Agricultural Biotechnology Centre, Murdoch University, Murdoch, Australia

**Correction: BMC Plant Biol 24, 262 (2024)**

10.1186/s12870-024-04987-9

Following publication of the original article [1], the authors identified minor typographical errors in the body of the article. The corrections were highlighted below in bold.

## Introduction

Incorrect line: A total of 100 g of groundnut oil contained 17.7 g of saturated fat, 48.3 g of monounsaturated (linoleic acid) fat, and 33.4 g of polyunsaturated (linoleic and linolenic acid) fat(https://ndb.nal.usda.gov/ndb/).

Correct line: A total of 100 g of groundnut oil contained 17.7 g of saturated fat, 48.3 g of monounsaturated **fat (primarily oleic acid)**, and 33.4 g of polyunsaturated **fat (comprising linoleic and linolenic acid**.

## Discussion

Incorrect line: Fatty acid desaturase is an important gene responsible for the conversion of linoleic acid to oleic acid,

Correct line: Fatty acid desaturase **enzyme catalyzes desaturation of oleic to linoleic acid**,

The authors overlooked and did not recognize these errors during manuscript writing and proofing stages. The original article [1] has been corrected.
